# The Teeth and the Feet

**Published:** 1892-07

**Authors:** Thomas C. Stellwagen


### THE TEETH AND THE FEET.[1]

BY THOMAS C. STELLWAGEN, M.D., D.D.S.[2]

The possibility of any practical connection between the teeth and the feet, up to the present time, does not appear to have attracted the attention of dentists in proportion to the importance that the gravity of the consideration demands. The guidance of fond parents in their responsible and delightful duties, the interest that they feel in the rearing and the welfare of their growing children, the aim and desire that their offspring should all be paragons of perfection and beauty, both physically and mentally, make the subject one of nearly universal interest.

The neglect of the correlation of these organs may be due to their remote separation in the human structure, being such as to divorce them from simultaneous study. Likewise it may be, in no small degree, the outcome of what may be denominated the fashionable drift of thought that diverts the general public from observation of the feet; except in the small class made by the dancing-masters and foot-ball team captains.

Among the disadvantages of so called modern progress, some of the most useful tradespeople are to be seen within their shops, neglected in the onrush. Fear may be expressed lest the hurry of the nineteenth century has resulted in stamping out of existence the true shoemaker and substituting in his stead the great shoe-factories. The modern luxuries of carriages and palace railway cars, of bicycles and tricycles, with their wonderful records of great distances traversed within enormously diminished periods of time, combine to revolutionize the use of the feet as means of and organs for propulsion. These causes operate in diverting the thoughts from the humble but wonderful locomotors; in effect, relegating them from the great practical purposes of life to the degrading duty of displaying costly adornments that deplete the purse and cripple the self-supporting powers of the wearers. We should draw a lesson from the famous battle of Larissa[3] in Thessaly, about the year 988, when Bohemond, leader of the Normans against the Greeks, was defeated by the latter aiming their shafts to kill the horses, thus making the dismounted riders easy victims with their unwieldy

---

[1] Read before the American Academy of Dental Science, February 3, 1892.

[2] Professor of Physiology, Philadelphia Dental College.

[3] Gibbon, vol. v. p. 474.

boots with toes two feet long, patterned after the form of scorpions. The tide of battle may be said to have turned upon the toes and heels of the Norman boots. Rarely has history more directly acknowledged the great importance of proper footwear. The deeds enacted by the feet upon the battle-fields were wonderful, where there hung in the balance such momentous affairs as revolutionized the thoughts and creeds of men, the very foundations of religious beliefs and moral enactments. By a stretch of imagination, the great and rapid marches of the German armies in the Franco-Prussian War, by inference somewhat distant and possible, may be accepted as an admission, although concealed from direct mention, that these troops must have been possessed of wonderfully good and useful feet.

Lest this thesis may be criticised for squandering too much of your valuable time, and paying too great tribute to the orthopedist's or chiropodist's functions rather than the odontologist's, it may be now asserted that the importance of the knowledge of how to properly care for the feet, as the basis of the whole bodily structure, will most profitably repay the specialists as well as the general practitioners of the healing art.

In the present era of science it is recognized by all who think of the philosophy of life-action that an ample supply of oxygen to each and every part of the living body is essential. By it the building of tissue, the storing and elimination of energy are brought about; upon it depends the no less practical functions for facilitating the economic removal and healthful disposal of the matters that otherwise would promptly become objectionable, effete, and often both harmful and dangerously threatening to life.

The micro-organic materials that in many diseases are abundant in the tissues of the body are now conceded to be most promptly and satisfactorily checked in their growth, formation, and multiplication by a luxuriant supply of vivifying and combining element, oxygen. The eradication of disease by destruction of the pathogenic microbes or their poisonous products and the abnormal cells of the tissues, the transformation of the multiform pathological germ-causes of sickness into harmless growths, are best performed and probably are only capable of accomplishment by the stimulation of the tissue-cells to their highest possible beneficial activities. These desirable changes are known to be responsive to such physical conditions as encourage and invite the lively mutation of combinations of the elements in the constructions and destructions of cell-substances, in obedience to the all-pervading laws of chemical affinity. The higher forms of life are well known to flourish when furnished with enormous quantities and constant supplies of this gas, for completion and perfection of the metabolic transitions that are as intrinsically necessary to the perfection of each cell as the whole body is dependent upon its individual parts for its composition and integrity.

That the microbes are the sole and entire cause of all pathological phenomena, and are to be held as entirely blamable for each and every disease, has been modified in many ways by others, who, if not more deep, are at least more recent thinkers. The ptomaines that are formed by these wicked and idle microbes, and the schizomycetes, with their iconoclastic or catabolic and destroying propensities, are set down as the principal evil influences. Other students of these phenomena of death and destruction liken them to thieving mobs that run riot through the communities of the legitimate citizen-cells and filch from the latter their hard-earned oxygen. The conditions of the tissues that permit the formation of the nests for these pernicious growths are impossible when healthy nourishment is the rule. From all sides the conclusion is forced upon us that when the natural and lavish supply of this important element is maintained in and throughout the remotest tissues, the symptoms and serious effects of most diseases are either in great part mitigated or entirely banished from those parts of the economy of nature.

In the light of modern scientific investigation, the various tubercular and glandular swellings that are pathognomonic of the scrofulous diathesis are produced by the caseation of matters, which, under strictly physiological operation of the anabolic and catabolic cell-changes, should have been cast out in the forms of the natural excretas by the proper depurative organs. The respiratory, renal, and dermal systems, and the lower portion of the alimentary canal furnish the principal parts of the complicated and as yet only partially understood apparatuses that are charged with the very important duty of elimination of the excretory matters from the blood. This is most perfectly and economically performed after each nutritive, component, proximate principle has by combustion set free at least the major part of the energies that were stored up in the processes of cell formation and growth. The natural energy, that is latent in the various organs, at proper time exhibits itself by the phenomena of their functional action. In these changes oxygen plays the chief rôle.

Failure to remove by these means of depuration the worn-out matter accumulated in the blood or the tissues is productive of disease. Even partial retentions and obstructions by these fecal matters in the alimentary canal are demonstrated often to be the precursory state that may be followed by a process of reabsorption and redistribution. The merest tyro in the healing art knows the dangers following these neglected duties that permit retention of matter that is more or less pernicious or violently poisonous. These products exert injurious influences and powers upon the tissues and cells that are bathed in them or assimilate them. By transmissions and harborings of these deleterious excretas in many tissues, the whole system is affected both directly and indirectly. Increase of the scavenger work to be accomplished in the communities of cells reduces the efficiency and the number of employés of the department of what might be compared to the public board of health in the commonwealth of the body.

The result of arrestation of the processes of digestion and absorption, even of healthy food, frequently is speedily followed by decomposition. Such degeneration of food-substances is most prompt at the temperature of the human body. This breaking down of the chemical compounds of the matters in the stomach and alimentary canal liberates gases that may in their turn be accountable for the painful sensations of distention of the containing portions of the digestive apparatus. The unnatural impingement and undue pressure upon their surroundings, together with the vast and unruly train of dyspeptic symptoms, follow. After centuries of suffering by untold millions of unfortunates, and expenditures of many more millions of money, in vain and often even unimaginable ways of research and attempt to discover a drug or remedy for the cure of this disease, the quest has been almost entirely unsuccessful. Like most human difficulties, it has been a simple, blind, and stupid one, raised by men's perverse disposition and stubborn persistence in the disregard and disobedience of the axiom of the divine command, "In the sweat of thy face shalt thou eat bread." (Genesis iii. 19.)

Physical labor, in a large majority of instances, is remarkably dependent upon the perfection of the adaptation of the feet to their very important and valuable purpose of serving as the foundations upon which the whole body is supported. The most superficial reference to architecture or construction will acquaint us with the rule that the perfection of a building, or superstructure, mainly depends upon the correctness of its foundation, in its adaptation to the support of its load.

The growth and development of the child is largely in proportion to the utility of its feet, and their capability of sustaining the various shocks and strains put upon them, which duty they should accomplish without undue fatigue or suffering. The passion young animals show for change of location, that is familiar to any who have noted the restlessness of children and that so frequently harasses and annoys the more elderly and sedate nurses or attendants, is but the natural outcome of the conservation and expenditure of nerve-force that, by its indulgence and exercise, stirs the muscular tissue to functional action, which is a powerful and natural cause of the healthful stimulation of the circulation of the blood. This, by reason of the wear and tear induced by use, causes an extra but not abnormal demand upon the digestive apparatus. In a word, it calls into play the various organs whose functions, when properly combined, only give forth harmonious interworking, and result in well-balanced development and growth.

No more thoroughly-accepted proposition can be quoted than that the restraint of the motions of young animals before they have attained their adult size and functions will cause them to be undersized, dwarfed, and often diseased and malformed. Observe the demonstration of this induction everywhere, and in all living voluntary moving beings. See how it is illustrated in the deficient hind limbs of large dogs that have been reared in close and confined quarters. This is shown where puppies have been kept chained to prevent them from injuring and destroying other animals or the surroundings of the houses, and disturbance of the households of their masters. An unnaturally peaked or pointed and elongated muzzle is found on such dogs, that may justly be said to speak louder than words, showing the corresponding lack of strength and breadth of both the maxillæ and their attached muscles.

Why go further than our daily experiences as dental practitioners for illustrations of these associations of causes and effects? Who are the children that we are obliged to treat for dental and oral irregularities? Do the unrestrained and healthy country-bred, barefooted urchins apply for the expanding apparatus for their dental arches? Is it not almost entirely prevalent among the miserable, dyspeptic, cramped, and tender-footed children who are unfortunate in the possession of parents and guardians, who have long been out of the line of common sense, and who are accustomed to outweigh the blessings of health and strength, by the morbid concern they evince for fashion and her proprieties? Does not the coveting of what is falsely termed social position make such rebels against children's rights persuade themselves that it is a true kindness to squeeze out of all semblance of shapely usefulness the soft and readily-mouldable cartilaginous bones, under the silly pretext of protecting their innocents from harm, and cold, and injury? The modern shoes seem especially designed and constructed with greatest ingenuity to antagonize the growth, the elasticity, the mobility, and the strength of the wearer's feet. By breaking down the arches, these devices deform, hopelessly cripple the toes, and cause painful corns, bunions, and other abnormities of growth. The circulation of the blood, the nourishment of the tissues, the resistance to cold, the power of nutrition in general, are these not all impaired by the shoes of the civilized child? Can we longer look on such cruelty practised in the name of humanity and parental love without loss of patience and raising our voices to abate the evil? Alas, we cannot stop here, the ankles are bound so tightly as to almost produce anchylosis; their functions are almost obliterated for these little sufferers. Not content with cruelty to the feet alone, now fashion aims to destroy the whole leg and even the knees. Little Lord Fauntleroy has cursed his poor imitators with leathern gaiters, stiff and awkward, heavy and tiresome. Oh, mothers put on balls and chains, out-Herod the Celestials, afflict your boys as well as your girls,—go beyond China!

Need it be asked, How can such a bestocked, hampered, weary, exhausted child develop and enlarge his lungs, have an appetite for bone- or muscle-making food that the very force required for its mastication will spread the alveolar processes, and produce healthful nutrition and growth of the mouth and its associated systems of glands, of muscles, of bones, of mucous membrane, of teeth?

The desperate criminal galley-slave can carry his ball and chain in his hand. The thief in the stocks is not doomed for all day and every day in the week. Both of these have attained their full growth and will not be made sickly and dwarfed. In what have these children so sinned that they must suffer such refinement of torture, as causes one to shudder at the thought of its being applied lightly and gently to the worst and lowest classes of men exclusively? Are the children thus prevented from giving you and their attendants trouble by the noise and clattering of their feet, and by occasionally escaping while nurse is ogled by a policeman?

Be consistent, arm your children's keepers with guns, tell them to shoot, to kill, if the little ones cross the dead-line; but in the name of all that is healthy and good, let them be at liberty within their play-grounds. Let them have at least as fair opportunities in their struggles with death, diphtheria, and denticulariæ, as the little urchin of the back alleys, who laugh at your pity for them in their bare feet, and take delight and pride in their designation "kid."

The practical side of this question for the orthodontist and the general odontologist should be to invite attention to the general system beginning at the feet as the foundation, and observe their adaptation to their work. If they or any portion of the locomotor apparatus are defective, it will greatly assist in the treatment of the mouth and of the teeth, if the part or parts of the body or limbs at fault be improved or corrected. Nature will be one of the most powerful means we can employ, even if only as an ally, in the stimulation of growth of the jaws. By proper exercise the appetite for suitable food will be begotten, and resulting from this will be practice in the use of the jaws in mastication. Then will awaken the dormant constructive action, and growth will be renewed.

The aim of this discourse is to emphasize a plea for the replacement of the morbid by healthy processes. It furthermore seems to be a proper question whether the very ingenious and complicated apparatus within the mouth, used for orthomorphia, has not cultivated too much reliance upon mechanical and too little regard for the natural means of correction. The presence of the apparatus for moving the teeth does not improve their masticatory functions, and although the expansion of the arches may be effected, still it might be assisted and hastened by the benefit derived from natural work. If the blood-flow be increased, nutrition of the bony support certainly will be a sequence of this functional activity. Chewing or biting upon a piece of hard wood, as recommended by the late Professor McQuillen, has often done more to expand arches and symmetrically arrange the teeth than whole drawers full of apparatus to be worn in the mouth and worked by springs, screws, and wedges. The flow of saliva that follows upon thought of sapid substances indicates the glandular action which results from a determination of the nutritive current to these parts, and is an illustration of the manner in which the control of secretions and growths are under nervous influences. Deprivation of pabulum to any part is quickly followed by sloughing. Diminution of the supply will result in proportionate arrestation of growth or development. If the lungs, the arms, the legs, or any portion of the economy is defective in size or texture, the most likely method of effecting repair of growth comes through judicious motion and increased use. Why then will not a law so universal act for the benefit of the jaws? If this be true, will not such exercise as provokes healthy desire for food of the kind qualified to build up and replace the consumed and worn-out tissues react upon the jaws through the increase of their nerves and blood-currents?

Some observations that your essayist has made upon those afflicted with crippled and clubbed feet seem to point in this direction; for, if the cases viewed, are not by some remarkable series of chances very exceptional, the crippling of the feet in an undue number of cases is accompanied or more likely followed by the deformity of the dental arches. Indeed, a curious confirmation of the proposition may be mentioned, that in a great many of the exceptions to the association of dental abnormities with impaired power of the feet, there were discovered to be well-marked vicarious auxiliaries for locomotion in strong hands and arms to wield canes and crutches.

The following table shows the proportion of regular and irregular mouths among a series of children having crippled or deformed feet.

| Boys. (*Over eight years.*) | |
|---|---|
| Regular | 2 |
| Slightly irregular | 8 |
| Irregular | 7 |
| Very irregular | 7 |
| Total | 24 |

| Boys. (*Eight years and under.*) | |
|---|---|
| Regular | 5 |
| Slightly irregular | 6 |
| Irregular | 1 |
| Total | 12 |

| Girls. (*Over eight years.*) | |
|---|---|
| Regular[1] | 8 |
| Slightly irregular | 6 |
| Irregular | 8 |
| Total | 22 |

| Girls. (*Eight years and under.*) | |
|---|---|
| Regular | 2 |
| Slightly irregular | 2 |
| Irregular | 2 |
| Total | 6 |

[1] One walked until thirteen years of age. One crippled at ten years of age. A few cases have been seen since reading the paper and are included above.

